# Self-Structuring in Water of Polyamidoamino Acids with Hydrophobic Side Chains Deriving from Natural α-Amino Acids

**DOI:** 10.3390/polym10111261

**Published:** 2018-11-13

**Authors:** Federica Lazzari, Amedea Manfredi, Jenny Alongi, Raniero Mendichi, Fabio Ganazzoli, Giuseppina Raffaini, Paolo Ferruti, Elisabetta Ranucci

**Affiliations:** 1 Dipartimento di Chimica, Università degli Studi di Milano, via C. Golgi 19, 20133 Milano, Italy; federica.lazzari@unimi.it (F.L.); amedea.manfred@unimi.it (A.M.); 2 Istituto per lo Studio delle Macromolecole (CNR), Via E. Bassini 15, 20133 Milano, Italy; mendichi@ismac.cnr.it; 3 Dipartimento di Chimica, Materiali ed Ingegneria Chimica “G. Natta”, Politecnico di Milano, via L. Mancinelli 7, 20131 Milano, Italy; fabio.ganazzoli@polimi.it

**Keywords:** polyamidoamino acid, self-structuring, pH-dependent circular dichroism, molecular dynamics, molecular simulations

## Abstract

This paper reports on synthesis, acid-base properties and self-structuring in water of chiral polyamidoamino acids (PAACs) obtained by polyaddition of *N*,*N*′-methylenebisacrylamide with *l*-alanine, *l*-valine and *l*-leucine (M-*l*-Ala, M-*l*-Val, M-*l*-Leu) with potential for selective interactions with biomolecules. The polymers maintained the acid-base properties of amino acids. In water, the circular dichroism spectra of PAACs revealed pH-dependent structuring in the range 3–11 and in the wavelength interval 200–280 nm. Taking as reference the values at pH 3, the differential molar ellipticities were plotted in the pH interval 3–11. Sigmoidal curves were obtained presenting inflection points at pH 8.1, 6.8 and 7.3 for M-*l*-Ala, M-*l*-Val and M-*l*-Leu, respectively, corresponding to the amine half-ionization. Theoretical modeling showed that PAACs assumed stable folded conformations. Intramolecular interactions led to transoid arrangements of the main chain reminiscent of protein hairpin motif. Oligomers with ten repeat units had simulated gyration radii consistent with the hydrodynamic radii obtained by dynamic light scattering.

## 1. Introduction

Chirality governs fundamental recognition and replication functions in biological systems, as for instance biomaterial-cell interactions [1,2]. Designing chiral synthetic polymers capable of self-assembling into stable secondary structures in water and exerting specific biorecognition represents an attracting biomimetic approach [3,4].

Leaving apart natural and synthetic polypeptides, many examples of bioinspired chiral polymers deriving from natural α-amino acids can be found in the literature [5]. Most of them, prepared by the radical polymerization of *N*-acryloyl- or *N*-methacryloyl-α-amino acids, are in fact polyacrylamides or polymethacrylamides bearing carboxylated chiral substituents on the nitrogen [6,7,8]. In other cases, the α-amino acid moieties are differently linked to polymethacrylate- [9], polyacrylate- [10], polyacetylene- [11,12], polyolefin- [13], polyvinyl ether- [14] and polyphosphazene- [15] backbones. These polymers show interesting pH-dependent solubility [16], self-assembly ability [17], and thermosensitivity properties [18,19]. In addition, they may show chirality-induced structuring in solution [7,12,13,20] and chiral recognition [14,21] capacities including differential interactions with organic cell components [21]. Chiral polypeptoids, that is, polypeptide analogues consisting of chiral *N*-alkyl-substituted polyglycines, behave in a similar way [22]. It may be observed that the preparation processes of the above polymers deprive the parent amino acids of amphoteric properties, in most cases by transforming their α-amine groups and, limited to polypeptides and polypeptoids, carboxyl groups into amide groups.

Recently, another family of α-amino acid-derived polymers, called polyamidoamino acids (PAACs), has materialized [23,24]. PAACs are prepared by Michael-type stepwise polyaddition of α-amino acids with *N*,*N*′-methylenebisacrylamide (MBA) in the same way as linear polyamidoamines (PAAs) [25]. The polymerization involves only the amino acid *prim*-amine group, which is transformed into a *tert*-amine group maintaining its basic properties. The amino acid carboxyl group is not involved. Therefore, PAACs are still endowed of the amphoteric properties peculiar of parent α-amino acids.

The first PAAC example, named *L*-ARGO7, employed *l*-arginine and MBA as monomers [23]. Subsequently, also the PAACs deriving from *d*-, and *d*,*l* arginine (*d*-, and *d*,*l*-ARGO7) were prepared and their structuring in aqueous media studied by circular dichroism spectroscopy (CD) and molecular modeling [24]. The CD spectrum of *d*,*l*-ARGO7 gave no evidence of structuring, whereas those of *d*- and *l*-ARGO7 showed negative- or positive ellipticity, respectively, with peaks at 228 nm indicating ordered secondary structures. Structuring proved strongly pH-dependent. Plotting differential molar ellipticity at 228 nm versus pH gave sigmoidal curves in mirror-image relationship with inflections at pH ~6.4, which, incidentally, corresponded to the half-ionization point of the chain amine nitrogen. The theoretical modeling of *l*-ARGO7 revealed intramolecular interactions leading to a relatively compact intramolecular self-organization of the polymer chain in water somehow reminiscent of the protein hairpin motif with extensive transoid arrangements of the main chain.

It was interesting to ascertain whether this peculiar behavior was typical of ARGO7 polymers, with their bulky and charged side substituent, or is a common feature of PAACs deriving from chiral α-amino acids. To this purpose, three novel PAACs obtained by polyaddition of MBA with natural α-amino acids carrying different hydrophobic substituents, namely *l*-alanine, *l*-valine and *l*-leucine, were prepared and studied. The aim of this paper is to report on the experimental and modeling results obtained for these new polymers.

## 2. Materials and Methods

**Materials.** Solvents and reagents, unless otherwise indicated, were analytical-grade commercial products and used as received. *l*-leucine (*l*-Leu, ≥98%), *l*-valine (*l*-Val, ≥98%,) and *l*-alanine (*l*-Ala, ≥98%) were purchased from Sigma-Aldrich (Milano, Italy), *N*,*N*’-methylenebisacrylamide (MBA, 96%) from Acros Organics (Milano, Italy) and lithium hydroxide monohydrate (LiOH·H_2_O, ≥98%) from Honeywell Fluka. 0.1 M Hydrochloric acid (HCl) and 0.1 M sodium hydroxide (NaOH) volumetric standard solutions were purchased from Fluka analytics (Milano, Italy). Distilled water (18 MΩ), purified by a Millipore Milli-Q apparatus, was used to prepare solutions and dissolve samples.

**Characterizations.** The ^1^H and ^13^C NMR spectra of PAACs were obtained in D_2_O at 25 °C using a Bruker Avance DPX-400 NMR spectrometer operating at 400.13 and 100.40 MHz (Bruker, Milano, Italy), respectively.

The molecular weights of PAACs were determined by Size Exclusion Chromatography (SEC). SEC traces were obtained with Toso-Haas TSK-gel G4000 PW and TSK-gel G3000 PW columns connected in series, using a Waters model 515 HPLC pump equipped with a Knauer autosampler 3800 (Knauer, Bologna, Italy), a Viscotek 270 light scattering/viscometric triple detector (Malvern, Roma, Italy), (LALS/RALS) and a refractive index detector (Waters, Model 2410). The mobile phase was a pH 8.00 ± 0.05 0.1 M Tris buffer solution with 0.2 M sodium chloride [26]. The operational conditions were: sample concentration 20 mg mL^−1^; flow rate 1 mL min^−1^; injection volume 20 µL; column dimensions 300 × 7.5 mm^2^, temperature 25 °C. The instrument optical constants were determined using PEO 19 kDa as a narrow polymer standard. All samples were filtered through a 0.2 µm syringe Whatman filter before measurements.

Dynamic light scattering (DLS) analyses were carried out on 0.5–20 mg mL^−1^ PAAC solutions in Milli-Q water, with a Malvern Zetasizer NanoZS instrument equipped with a laser fitted at 532 nm (Malvern, Roma, Italy), and fixed 173° scattering angle. Before analysis, samples were filtered through a 0.2 µm syringe Whatman filter. The pH was adjusted to the selected value, using 0.1 M HCl or 0.1 M NaOH aqueous solutions. Measurements were performed in triplicate, and the reported values are averaged over of 10 runs.

Circular Dichroism (CD) spectra were obtained using a JASCO J-500CD spectrometer (Jasco, Lecco, Italy), by scanning from 200 to 300 nm in a 1 cm path-length quartz cell at 50 nm min^−1^ scan speed. 0.5 mg mL^−1^ PAAC solutions in 0.1 M NaCl were analyzed in the presence or absence of denaturating agents. The pH was adjusted with 0.1 M HCl or 0.1 M NaOH aqueous solutions and measured by a combined Metrohm microelectrode. Before recording spectra, samples were thermostated for 1 h at the desired temperature. CD spectra were normalized on the basis of the molar concentration of the repeating units (≈1.83 ± 0.17 mM) and reported as Molar Ellipticity (*θ* expressed as mdeg M^−1^ cm^−1^). Measurements were reported as the average of 3 runs.

The acid-base properties of PAACs were determined on 0.05 M solutions, referred to the repeat units, in 0.1 M NaCl aqueous solutions in nitrogen atmosphere and at 25 °C. Samples were potentiometrically titrated forward with 0.1 M NaOH and backward with 0.1 M HCl. In forward titrations, the pH was previously adjusted to 1.6–1.8 with 1.0 M HCl, while in backward titrations pH was previously adjusted to pH = 11.8–12.0 with 0.1 M NaOH. The pH-meter, a Metrohm-Primatrode with NTC electrode connected to a 827 pH lab Metrohm, was calibrated against two pH standard buffers thermostated at 25 °C. All titration experiments were performed in quadruplicate. The resulting titration curves were analyzed as described in the Supplementary Materials.

**Synthesis of PAAC. *M*-*l*-*Ala***. *L*-alanine (2.27 g, 25.00 mmol) and MBA (4.02 g, 25.00 mmol) were suspended in water (8 mL). LiOH·H_2_O (1.07 g, 25.00 mmol) was then added to deprotonate the amine groups. The resultant slurries were heated to 50 °C and magnetically stirred until homogeneous. Stirring was then discontinued and the reaction mixtures maintained at 50 °C for 6 days with occasional shaking. After this time, the reaction mixture was acidified to pH 3.5 with 6 M hydrochloric acid and ultrafiltered through a membrane with nominal molecular weight cut-off 100,000 to eliminate possible particulate impurities. The passed-through portion was then further ultrafiltered through a membrane with nominal cut-off 5000. The product, an off-white powder, was then retrieved from the retained portion by freeze-drying. Yield 81%.

$M_{w}$ = 7800; $M_{w}/M_{n}$ = 1.44. ^1^H NMR (D_2_O, 400.132 MHz, ppm): *δ* 1.40–1.43 (m, 3H, C**H_3_**CH), 2.64–2.79 (m, 4H, COC**H_2_**), 3.24–3.42 (m, 4H, C**H_2_**N), 3.90–3.96 (m, 1H, C**H**COO^−^), 4.54 (s, 2H, NHC**H_2_**NH). ^13^C NMR (100.623, D_2_O, ppm): *δ* 15.14, 33.31, 44.19, 46.35, 61.48, 175.16, 181.28.

***M-l-Val*** was prepared as M-*l*-Ala by substituting *l*-valine (2.99 g, 25.00 mmol) for *l*-alanine. Yield 47%. $M_{w}$ = 5600; $M_{w}/M_{n}$ = 1.30. ^1^H NMR (D_2_O, 400.132 MHz, ppm): *δ* 0.92–0.93 (d, 3H, (C**H_3_**)_2_CH), 1.04–1.05 (d, 3H, (C**H_3_**)_2_CH), 2.23–2.25 (m, 1H, (CH_3_)_2_C**H**), 2.67–2.75 (m, 4H, COC**H_2_**), 3.44–3.50 (m, 5H, C**H_2_**N and C**H**COO^−^), 4.53 (s, 2H, NHC**H_2_**NH). ^13^C NMR (100.623, D_2_O, ppm): *δ* 17.23, 19.37, 25.85, 28.82, 44.23, 47.28, 73.43, 170.94, 172.52.

***M-l-Leu*** was prepared as M-*l*-Ala by substituting *l*-leucine (3.35 g, 25.00 mmol) for *l*-alanine. Yield 59%. $M_{w}$ = 6200; $M_{w}/M_{n}$ = 1.51. ^1^H NMR (D_2_O, 400.132 MHz, ppm): *δ* 0.89 (t, 6H, C**H_3_**CH), 1.50–1.55 (m, 1H, **CH**(CH_3_)_2_), 1.76–1.83 (m, 2H, C**H_2_**CH), 2.72–2.75 (m, 4H, COC**H_2_**), 3.42–3.45 (m, 4H, C**H_2_**N), 3.69–3.73 (m, 1H, C**H**COO^−^), 4.54 (s, 2H, NHC**H_2_**NH). ^13^C NMR (100.623, D_2_O, ppm): *δ* 15.14, 33.31, 44.19, 46.35, 61.48, 175.16, 181.28.

All ^1^H-NMR spectra are reported in Figure S1 (Supplementary Materials). The FTIR-ATR spectra, recorded according the procedure described in the Supplementary Materials, are reported in Figure S2.

## 3. Results and Discussion

### 3.1. Synthesis of Polyamidoamino Acids

**Scheme 1:**
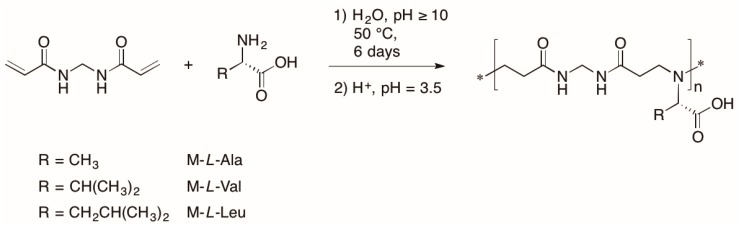
Synthesis of PAACs.

The PAACs studied in this work were prepared in water by Michael-type polyaddition of natural α-amino acids with *N*,*N*′-methylenebisacrylamide (MBA) (Scheme 1). The same synthetic method is normally adopted, at room temperature, for preparing linear polyamidoamines (PAAs) from prim-amines or bis-*sec*-amines and bisacrylamides. α-Amino acids other than glycine were for a long time considered unsuitable to this purpose owing to the very low reactivity of α-amines in the second addition step with bisacrylamides under the above conditions. However, in the synthesis of ARGO7 [23] this difficulty was overcome by raising the reaction temperature and prolonging the reaction time, a device that with PAAs often lead to partial hydrolytic degradation of the newly formed PAA chains [27]. With ARGO7, this inconvenience was significantly reduced, a fact tentatively ascribed to the amphoteric nature of the polymer exerting a buffering action in the surrounding micro-environment [24]. It was envisaged that this could hold true also for natural α-amino acids structurally simpler than arginine. In the present work, the polyaddition of MBA with *l*-alanine, *l*-valine and *l*-leucine was carried out in water at 50 °C and pH ≥ 10 for 6 days (Scheme 1). In no cases, vinyl radical polymerization that, incidentally, would have produced insoluble cross-linked matter, was observed. On the other hand, some hydrolytic degradation might have taken place, but it was too small to be detected by NMR. Samples of the corresponding PAACs named M-*l*-Ala, M-*l*-Val and M-*l*-Leu with weight-average molecular weight 7800, 5600 and 6200 and polydispersity index 1.44, 1.30 and 1.51, respectively, were obtained and their physicochemical properties and structuring ability in water studied.

### 3.2. Acid-Base Properties

**Figure 1:**
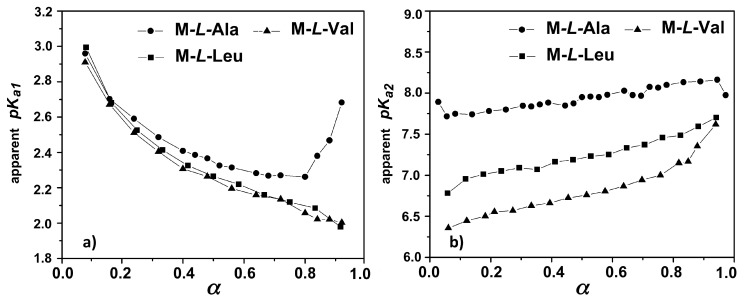
Dependence of PAAC apparent (**a**) carboxyl- and (**b**) amine *pK_a_* values on the dissociation degree. Operational conditions: 0.05 M repeating units in aqueous 0.1 M NaCl at 25 °C.

Similar to their parent α-amino acids, PAACs contain carboxyl- and amine groups and, therefore, are amphoteric polyelectrolytes. Being polyelectrolytes, their “apparent” *pK_a_*_1_ (carboxyl groups) and *pK_a_*_2_ (*tert*-amine groups) constant values depend on the dissociation degree *α*. This dependence was ascertained by plotting the *pK_a_*_1_ and *pK_a_*_2_ values calculated from the Henderson-Hasselbalch equation (Equation (1)) versus *α* (Figure 1).

(1) $$pH=pK_{a}-\log\frac{1-\alpha }{\alpha }$$

In Equation (1) *K_a_* is the apparent weak acidic dissociation constant of the group being pH-determining in the buffer titration zone considered.

Interestingly, the trends of both apparent *pK_a_*’s versus *α* are opposite to those normally encountered in polymeric acids and bases [28], in which as the ionization degree increases, that is, the polymeric ion becomes more strongly charged, a greater amount of work is required to remove a proton from a carboxyl group or to add a proton to an amine group owing to charge repulsion. By contrast, the “apparent” *pK_a_*_1_ values of all PAACs decrease by increasing the ionization degree in the *α* range 0.1–0.8 for M-*l*-Ala and 0.1–0.9 for M-*l*-Val and M-*l*-Leu, whereas in all cases the apparent *pK_a_*_2_ values increase in the *α* range 0.05–0.95 (Figure 1b). In this respect, as regards *pK_a_*_1_, the PAAC behavior within the above pH ranges approaches that of polymeric carboxylic acids, whose deprotonation in the presence of cationic polyelectrolytes is promoted by the formation of strong inter-polyelectrolyte complexes [29]. It was postulated that the shielding of the anionic groups of a polymeric carboxylic acid by an oppositely charged polyelectrolyte was more effective than that of the same polymeric carboxylic acid with an oppositely charged monovalent electrolyte. In PAACs strong polyelectrolyte intramolecular interactions may be established between neighboring groups with opposite charges, possibly favored by the PAAC conformational compactness in solution (see below). Similarly, the proton release by ammonium groups involves breaking polyelectrolyte complexes with carboxylate groups, thus increasing the apparent *pK_a_*_2_ value.

It may be observed that, in case of M-*l*-Ala, at *α* > 0.8 the ionization equilibrium of carboxyl groups deviated from that of the same PAAC at lower *α* values, approaching the normal behavior of polymeric acids [29]. This is probably due to the fact that the α-substituent of alanine is the least bulky and hydrophobic of the series. Probably, at high ionization degrees M-*l*-Ala is highly hydrated and, consequently, the electrolytic interactions between oppositely charged neighboring groups loosen.

The *pK_a_*_1_ and *pK_a_*_2_ values reported in Table 1 were determined by potentiometric titration from the half-neutralization points, where *pH* = *pK_a_*, following the procedure described in the Supplementary Materials (Figure S3 and Table S1). For *α* ≠ 0.5, polyelectrolytes usually follow the modified Henderson-Hasselbalch equation (Equation (2)).

(2) $$pH=pK_{a}-\beta \log\frac{1-\alpha }{\alpha }$$

where *K_a_* is the apparent weak acidic dissociation constant of the group being pH-determining in the buffer titration zone considered and *β* is the Katchalsky and Spitnik parameter [30] accounting for possible interactions between ionizable groups of repeat units being spatially or topologically adjacent. *β* values (Table 1) were determined by following the procedures described in the Supplementary Materials (see also Figure S4).

**Figure 2:**
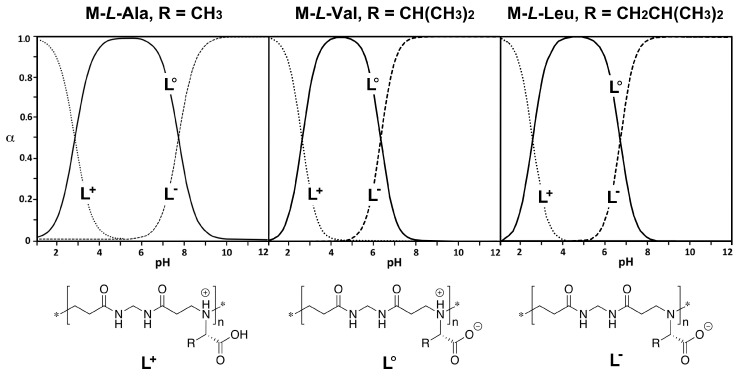
Speciation diagrams of M-*l*-Ala, M-*l*-Val and M-*l*-Leu.

The PAAC repeating units can exist in three ionization states, whose distribution versus pH were determined from the *pK_a_* and *β* values reported in Table 1 following methods described in the Supplementary Materials. The related diagrams are shown in Figure 2.

### 3.3. Circular Dichroism Spectroscopy

**Figure 3:**
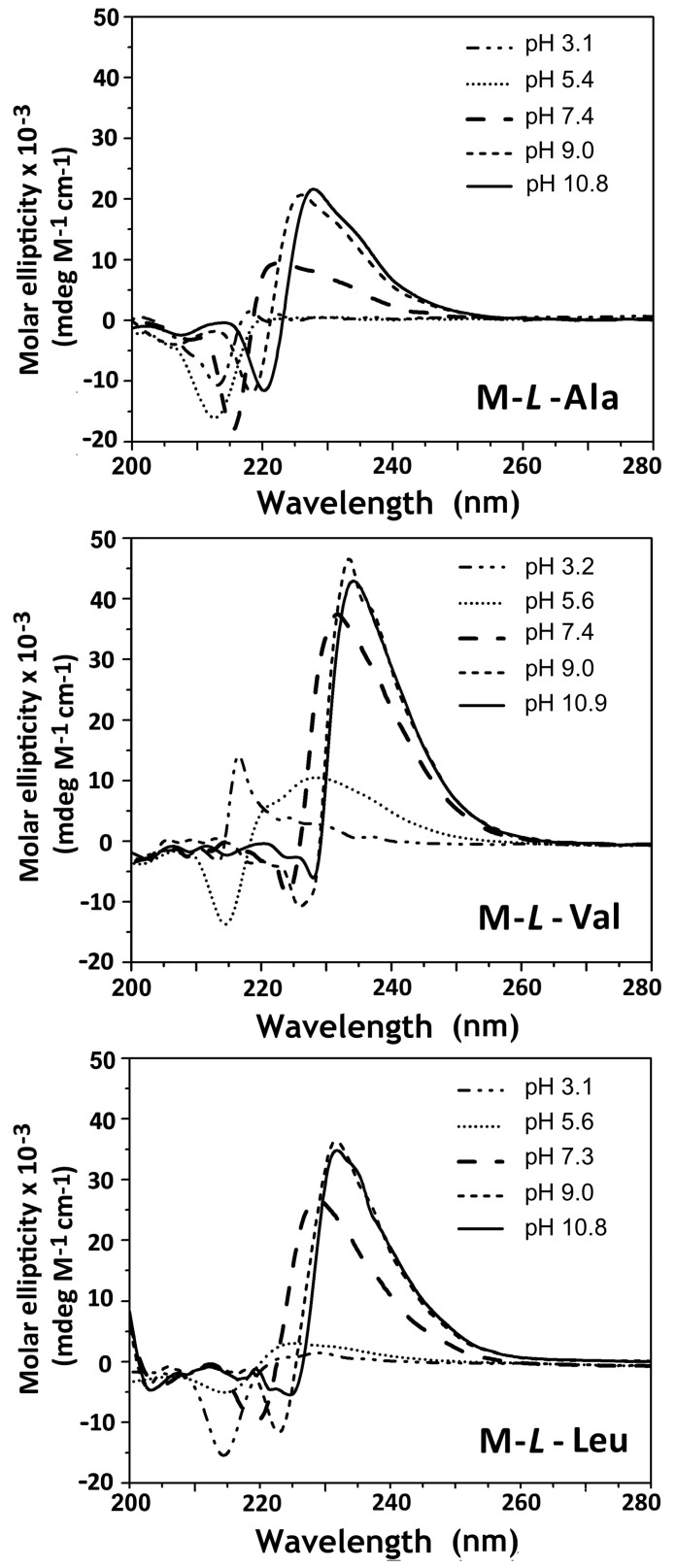
pH Dependence of PAAC CD spectra. Operational conditions: 1.83 ± 0.17 mM repeating units in 0.1 M NaCl solution, 25 °C. For the sake of clarity, some curves have been omitted and reported in Figure S5.

The CD spectra of all PAAC samples in 0.1 M NaCl showed clear evidence of pH-dependent structuring in the pH range 3–11 and in the wavelength interval 200–280 nm (Figure 3 and Figure S5), suggest that this property, already observed for *d*- and *l*-ARGO7, the first chiral PAACs, might be general for all chiral members of the family. At pH ≥ 7, all spectra show a positive molar ellipticity peak (referred to PAAC repeat units) with maximum centered at 228 (M-*l*-Ala), 234 (M-*l*-Val) and 231 (M-*l*-Leu) nm. The maximum value is pH-dependent and, in all cases, lowest for M-*l*-Ala.

**Figure 4:**
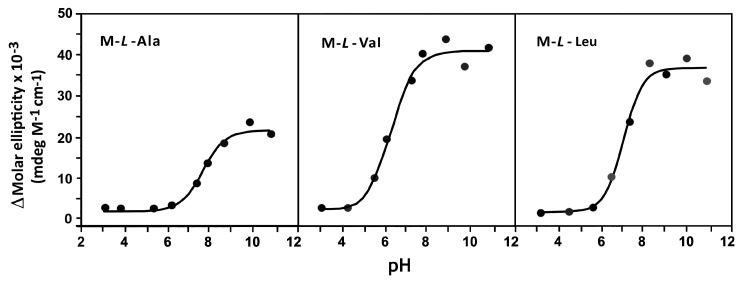
pH Dependence of M-*l*-Ala, M-*l*-Val and M-*l*-Leu differential molar ellipticity at 228, 234 and 231 nm, respectively. Operational conditions: 1.83 ± 0.17 mM repeating units in 0.1 M NaCl solution. Based on data points reported in Figure S5.

Plotting between pH 3 and 11 the differential molar ellipticity calculated taking as reference the values at pH 3, sigmoidal curves are obtained presenting for M-*l*-Ala, M-*l*-Val and M-*l*-Leu, respectively, a lower plateau in the pH intervals 3.0–5.5, 3.0–4.8 and 3.0–5.0; an inflection point at pH 8.1, 6.8 and 7.3; and an upper plateau at pH ≥ 10.4, pH ≥ 9 and pH ≥ 9.9 (Figure 4). The speciation diagrams (Figure 2) explain these results. The strongly acidic carboxyl groups are highly de-protonated, hence negatively charged from pH 4 upwards. The weakly basic *tert*-amine groups are highly protonated, hence positively charged, in the first plateau region but nearly completely de-protonated in the upper plateau region. For each PAAC, the sigmoid inflection point corresponds to the half-deprotonation of its amine groups, that is, occurs at *pH* = *pKa*_2_ (see Table 1). Apparently, the protonation degree of the chain amine groups as a function of pH is the main factor governing structuring and, consequently, the shape of the CD spectrum of PAACs in water.

At pH values >9 and <3, for all PAAC samples, the positive ellipticity is accompanied by a negative ellipticity always positioned at lower wavelengths respect to the main positive peak. Its position shifts to lower wavelengths by lowering pH. The negative peak has a minimum at approximately the same pH of the maximum of the related positive peak, but its absolute intensity is always lower.

**Figure 5:**
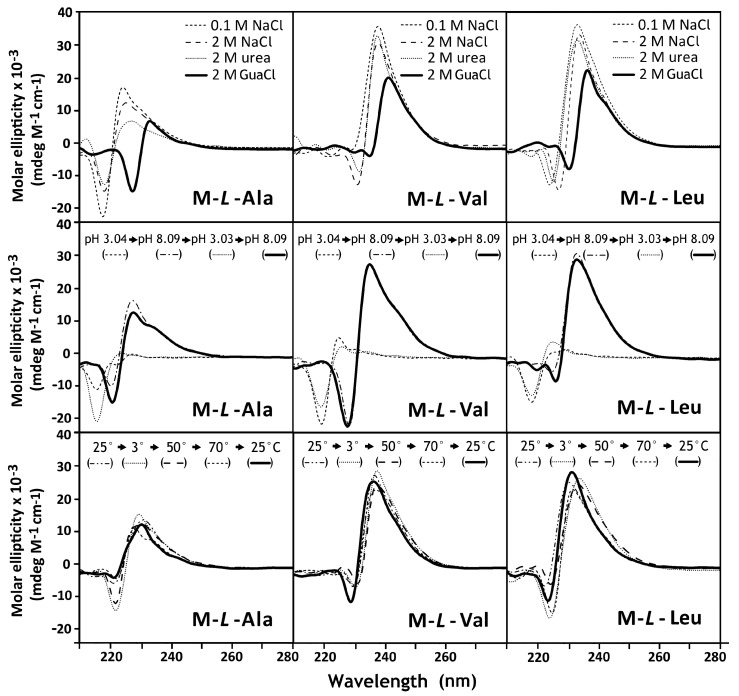
Dependence of PAAC CD spectra on a change of: ionic strength and presence of denaturating agents, namely urea and guanidinium chloride (GuaCl) (upper row); pH (intermediate row); temperature (bottom row). Operational conditions: 1.83 ± 0.17 mM repeating units and, where not otherwise stated, in 0.1 M NaCl solution.

The spectra of all PAAC samples are only modestly affected by ionic strength (0.1 M and 2 M sodium chloride) and, for M-*l*-Val and M-*l*-Leu, by 2 M urea (Figure 5). The CD spectra reveal that for M-*l*-Ala, M-*l*-Val and M-*l*-Leu structuring, once formed at a given pH, is quickly and reversibly modified by pH changes (Figure 5). The same holds true for temperature in the interval 3–70 °C, to a far lesser degree for M-*l*-Val and M-*l*-Leu, but not for M-*l*-Ala, in whose CD spectrum at 70 °C the negative ellipticity nearly vanishes (Figure 5). It may be reminded that also the CD spectra of the first chiral members of the family, *d*- and *l*-ARGO7, showed the same resistance to temperature, ionic strength and urea [24]. However, they were also nearly insensitive to 2 M guanidinium chloride, possibly due to the presence of a guanidine pendant in their repeat unit, whereas the spectra of M-*l*-Ala, M-*l*-Val and M-*l*-Leu are not. In the presence of guanidinium chloride, in the M-*l*-Ala spectrum both positive and negative peaks are shifted toward higher wavelengths and their absolute intensities reduced. The same occurs for M-*l*-Val and M-*l*-Leu, but to a lesser extent.

### 3.4. Dynamic Light Scattering (DLS) Measurements

**Figure 6:**
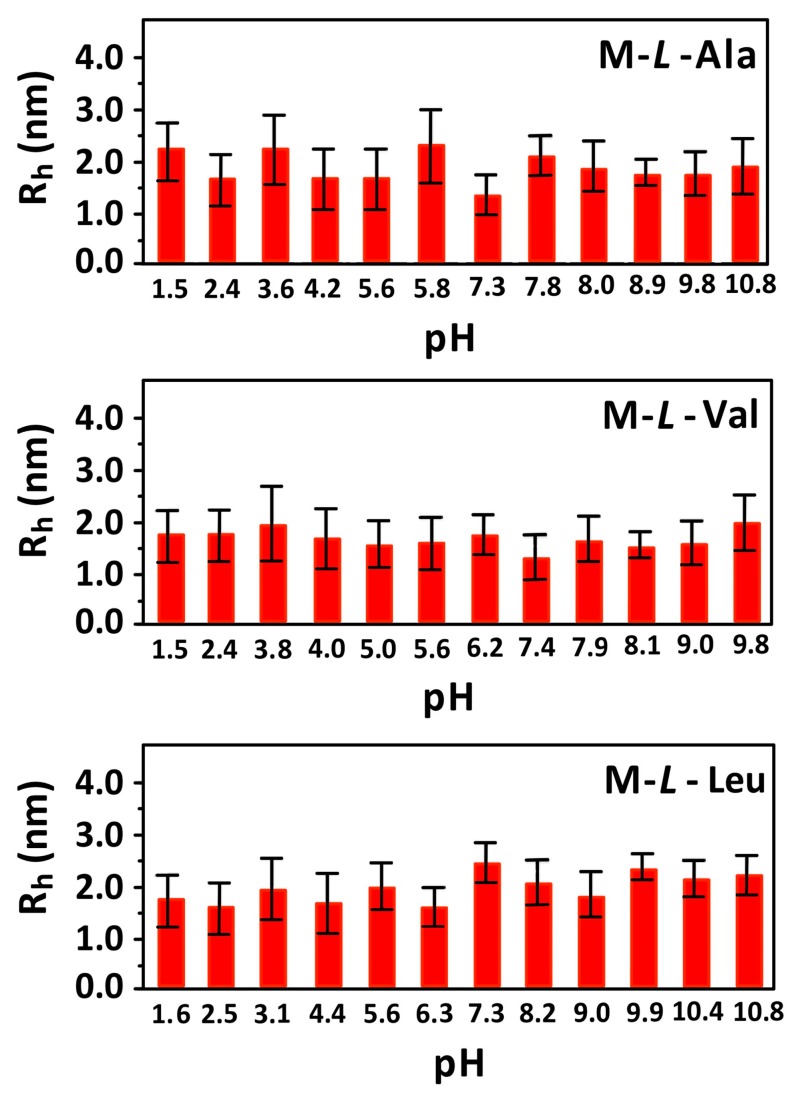
pH Dependence of PAAC hydrodynamic radius from DLS at 25 °C in 0.1 M aqueous NaCl. Polymer concentration 1 mg mL^−1^.

**Figure 7:**
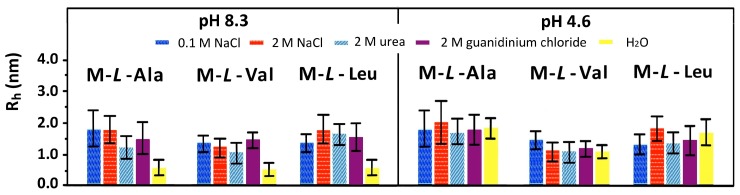
PAAC hydrodynamic radii from DLS in: 0.1 M and 2 M NaCl, 2 M urea, 2 M guanidinium chloride and plain water. Operational conditions: polymer concentration 1 mg mL^−1^, 25 °C.

The PAAC hydrodynamic radii, *R_h_*, obtained in 1 mg mL^−1^ solutions in aqueous 0.1 M NaCl by DLS measurements did not significantly vary within a wide pH range (1.5–11) notwithstanding their amphoteric nature (Figure 6). The particle size was stable at least up to one month suggesting that their compact structure could be ascribed to intramolecular interactions. The effect of ionic strength and denaturating agents was investigated (Figure 7). Both factors did not significantly affect *R_h_* both at acidic and basic pH’s, apart from pH 8.3 water, where the dimensions of all PAACs were significantly lower, probably because in water additional intramolecular interactions existed, which were loosened in the presence of salts or denaturating agents.

**Figure 8:**
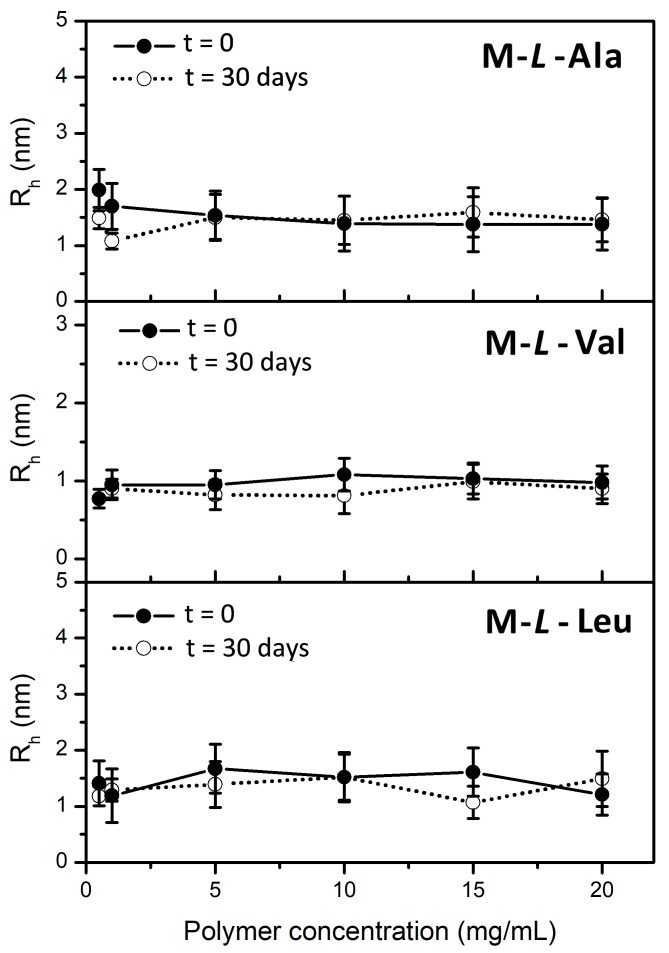
Concentration dependence of PAAC hydrodynamic radius in Milli-Q water pH 4.74 ± 0.72 at 0 and 30 days.

The negligible *R_h_* dependence on concentration, as shown for instance in Milli-Q water in the 0.5–20 mg mL^−1^ range (Figure 8) suggested that significant intermolecular aggregation in aqueous media did not occur and the polymers intramolecularly self-assembled into single chain nanoparticles.

### 3.5. Theoretical Modeling

Adopting a simulation protocol used in previous work [31,32,33,34], molecular dynamics (MD) simulations at room temperature and final energy minimizations using molecular mechanics (MM) methods were carried out for M-*l*-Ala, M-*l*-Val, M-*l*-Leu with ten repeat units (see Scheme 1) bearing a positive (L^+^), a null (L^0^) and a negative (L^−^) charge per repeat unit (see Figure 2 and Table 2), using the same strategy used in the previous theoretical study for the PAAC from *l*-arginine, *l*-ARGO7 [24]. More details about the adopted software and force field, and about the simulation protocol are reported in the Supplementary Materials. In particular, starting from a fully elongated chain, at first the conformational properties of PAACs in an effective dielectric medium were investigated, and then more extensive simulation runs in the explicit presence of water molecules adopting a periodic simulation box were carried out. In the presence of water, after a preliminary molecule energy minimization in the chosen environment, the MD runs were performed at room temperature with final geometry optimization after achieving equilibrium. In both simulation environments a similar conformational behavior of the polymer chains was found, never displaying the typical polyelectrolyte extended conformations, in keeping with what already found for *l*-ARGO7 [24]. Some MD runs were also carried out for M-*l*-Ala at pH 1 using a different strategy starting again from a fully elongated chain in the effective dielectric medium. Briefly, MD runs were first performed at 500 K, then the temperature was decreased to 400 K and eventually to 300 K to check for possible trapping in a metastable state and to test for the robustness of the final conformation. No significant differences to within 4% were found for the calculated size descriptors reported below (Figure S6). For a meaningful comparison with the experimental results, the following discussion will be limited to the simulations carried out in explicit water.

As initial geometry, a fully extended conformation characterized by 8.45 nm end-to-end distance was considered for all PAACs. After the simulation runs, the polymer chains displayed coiled structures at all pH values considered, as quite evident from the molecular sizes reported in Table 2.

**Figure 9:**
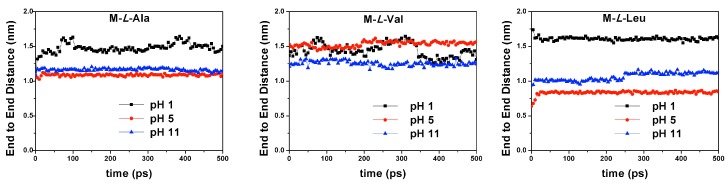
Time dependence of PAACs’ end-to-end distance within the MD run at different pH values.

In particular, a nearly spherical shape was observed both at pH 5 and pH 11, where the chain conformations were more compact than at pH 1 (see the values of the gyration radius and of the surface accessible to the solvent in the Table 2, the latter quantity being defined as the surface accessible to a spherical probe having a 0.14 nm radius, roughly the size of a water molecule). At pH 1, the chains showed larger size (Table 2) and a significantly more anisotropic molecular shape. These coiled structures were quickly achieved in the MD runs, with small fluctuations after equilibration, as shown for instance by the end-to-end distance values versus the simulation time in the explicit solvent for the three polymers (Figure 9).

**Figure 10:**
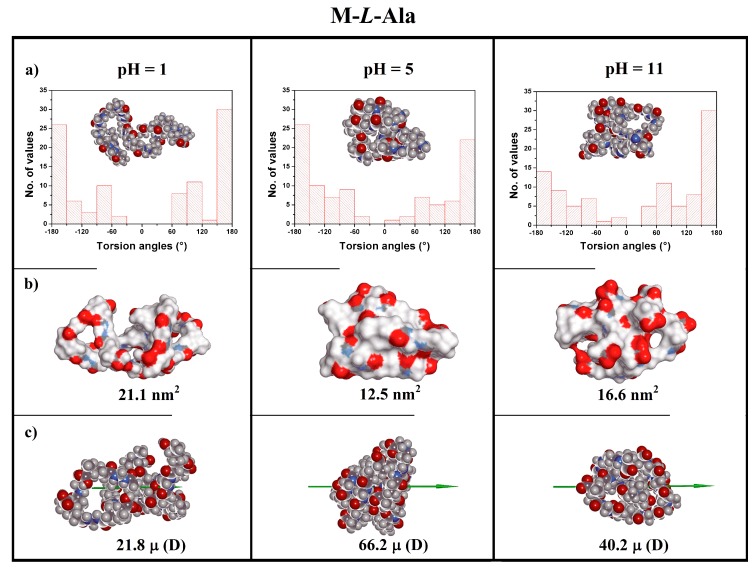
(**a**) Main chain molecular conformations in water at the end of the MD runs for M-*l*-Ala at pH 1, 5 and 11 and torsion angle distributions around the main chain bonds: the histogram are shown with a binning of 30°. Color codes: C atoms dark grey; H atoms light gray; N atoms blue; O atoms red. (**b**) Solvent accessible surface area in water. The surface area is in dark grey near C atoms, in light gray near H atoms, in blue near N atoms and in red near O atoms. (**c**) Dipole moments in water. Color codes are the same as in row (**a**).

**Figure 11:**
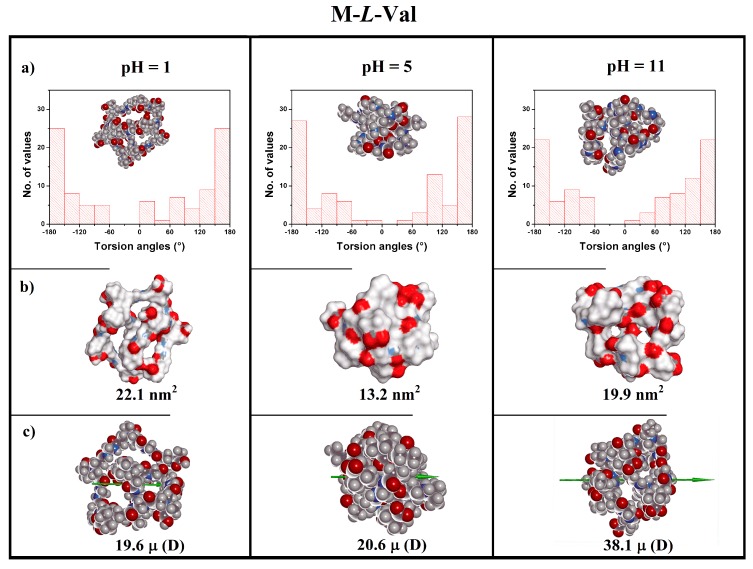
(**a**) Main chain molecular conformations in water at the end of the MD runs for M-*l*-Val at pH 1, 5 and 11 and torsion angle distributions around the main chain bonds: the histogram are shown with a binning of 30°. Color codes: C atoms dark grey; H atoms light gray; N atoms blue; O atoms red. (**b**) Solvent accessible surface area in water. The surface area is in dark grey near C atoms, in light gray near H atoms, in blue near N atoms and in red near O atoms. (**c**) Dipole moments in water. Color codes are the same as in row (**a**).

**Figure 12:**
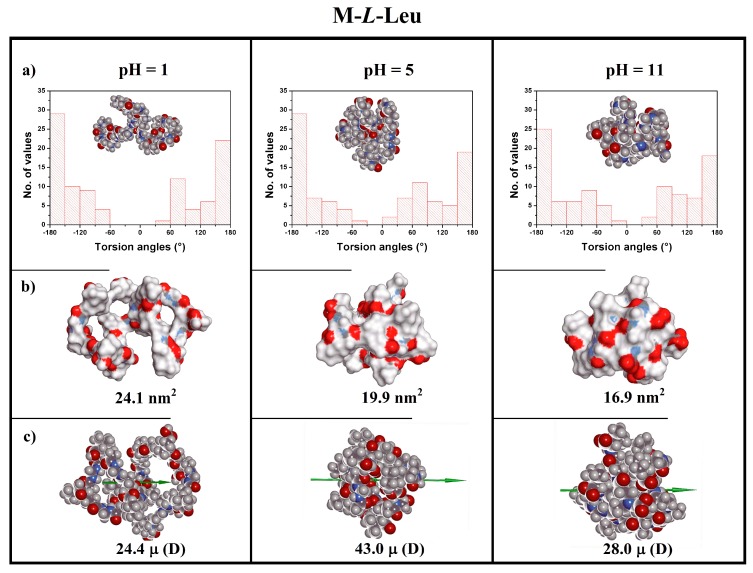
(**a**) Main chain molecular conformations in water at the end of the MD runs for M-*l*-Leu at pH 1, 5 and 11 and torsion angle distributions around the main chain bonds: the histogram are shown with a binning of 30°. Color codes: C atoms dark grey; H atoms light gray; N atoms blue; O atoms red. (**b**) Solvent accessible surface area in water. The surface area is in dark grey near C atoms, in light gray near H atoms, in blue near N atoms and in red near O atoms. (**c**) Dipole moments in water. Color codes are the same as in row (**a**).

At any pH, the *R*_g_ showed little dependence on the amino acid hydrophobic side groups, as shown in Table 2, suggesting that the coil size is dictated by the conformational properties of the backbone and, to a minor extent, by the protonation state of carboxylate moiety and of the amine group, with a relatively minor dependence from the overall molecular charge. As already found for *l*-ARGO7, for all PAACs the simulations in water showed slightly larger sizes than in the dielectric medium, particularly so at pH 1 due to the explicit hydration producing in all cases a somewhat larger molecular size and a less spherical shape. At pH 1 a somewhat loose, highly hydrated intramolecular organization was found, as evident from the solvent accessible surface values (Figure 10, Figure 11 and Figure 12). In particular, the large cavities present in this surface allowed for a very efficient molecular hydration in spite of the relatively compact conformation.

The simulated *R_g_* values of PAACs bearing a positive (L^+^), a null (L^0^) and a negative (L^−^) charge per repeat unit corresponding to different pH values (see Table 2), were compared with the *R_h_* values obtained from DLS measurements for the same overall charges with polydisperse PAAC samples (Table 3).

The compact PAAC structures in solution predicted by MD were fully consistent with the experimental results of Table 3 obtained for polydisperse samples of higher molecular weight, taking into account the different calculated radii (i.e., *R_g_* and *R* vs. *R_h_*) and the standard deviation of the experimental data. In addition, the theoretical values at pH 1 and 5 and the experimental data at pH 1.5 and 5.0 did not reveal significant differences. The experimental values at pH 9.0 were not comparable with those calculated at pH 11. On the other hand, DLS measurements at basic pH’s in Milli-Q water, performed without equating the ionic strength by adding NaCl, approached the instrument detection limits, and measurements at pH > 9.0 were unreliable.

The molecular conformation leading to the compact molecular size described above was dictated by the intramolecular interactions due to the charged or polar groups and by the hydrophobic interaction among the alkyl residues. In the MD run, the PAAC chains showed a rigidity similar to that previously shown by *l*-ARGO7 [24]. As with *l*-ARGO7, the intramolecular interactions led to a few folded hairpin-like conformations [24], as evidenced in Figure 10, Figure 11 and Figure 12 by roughly straight and antiparallel strands. These hairpin motifs were characterized by a typical distribution of torsional angles (defined by a sequence of four consecutive atoms) around the main chain bonds. In the case of positively charged and zwitterionic repeat units, torsion angles were mostly found in transoid arrangements peaked around ±180° (Figure 10, Figure 11 and Figure 12), while values in the ±60° to ±120° range allowed for strand turns and random conformations. A somewhat less pronounced transoid arrangement of the chain backbone was found with negatively charged repeat units, owing to local large repulsive intermolecular interactions among carboxylate groups.

Finally, the pH dependence of the overall dipole moment *μ* (Table 2) is consistent with the pH dependence of the CD spectra (Figure 3). As previously found for *l*-ARGO7, for PAACs *μ* was lowest at pH 1, where all units were positively charged (Figure 2) and many local dipole vectors pointing to opposite directions mutually compensate. Furthermore, for M-*l*-Ala and M-*l*-Leu the dipole moment was highest at pH 5, where charges were electrically balanced. It moderately decreased at pH 11, where most units were negatively charged, and the local dipoles largely depended on the carboxylate groups (Figure 10, Figure 11 and Figure 12). Conversely, for M-*l*-Val the dipole moment was highest at pH 11 with a negatively charged repeat unit due to a combination of charged groups and local dipoles associated with the chemical bonds.

## 4. Conclusions

A small library of new PAACs was synthesized by Michael-type polyaddition of MBA with *l*-alanine, *l*-valine and *l*-leucine, and the acid-base properties as well as the self-structuring in aqueous solution of the resultant polymers were studied. PAACs are amphoteric, since in their preparation the properties of the parent amino acid amine- and carboxyl groups are preserved.

In water, the PAACs considered showed CD spectra revealing, in the wavelength interval 200–280 nm, a clearly pH-dependent self-structuring. The differential molar ellipticities, calculated adopting the values at pH 3 as reference and plotted versus pH in the interval 3–11, gave sigmoidal curves with lower plateaus at acid pH and upper plateaus at basic pH, with inflection points corresponding to the half-neutralization of the amine groups, that is, pH 8.1, 6.8 and 7.3 for M-*l*-Ala, M-*l*-Val and M-*l*-Leu, respectively. The pH induced conformational changes were quickly reversible by changing pH. In the case of M-*l*-Ala, the CD spectra were remarkably sensitive to denaturating agents, in particular guanidinium chloride, and temperature above 70 °C. The CD spectra of M-*l*-Val and M-*l*-Leu were less, but still appreciably sensitive to the same agents.

DLS measurements in 0.1 M NaCl gave hydrodynamic radii stable at 25 °C for at least 1 month and unaffected by pH in the range 1–11, ionic strength up to 2 M NaCl, 2 M guanidinium chloride and sample concentration in the range 1–20 mg mL^−1^. These data suggested that PAACs intramolecularly self-assembled into single chain nanoparticles.

Theoretical modeling studies carried out on PAAC oligomers with ten repeat units showed stable folded conformations due to intramolecular interactions leading to main chain arrangements reminding hairpin motif of proteins. The simulated gyration radii of the same oligomer did not vary with the size of the hydrophobic side substituents were consistent with the hydrodynamic radii obtained by DLS measurements on polydisperse samples of higher molecular weight.

As a final conclusion, PAACs with hydrophobic side chains share with ARGO7 stereoisomers the ability to self-structure in water [24]. Coupling chirality with multifunctionality and pH-sensitiveness, these polymers may open interesting perspectives in selective interaction with chiral molecules, for instance in the biomedical field.

## Figures and Tables

**Table 1:** *pK_a_* and *β* values of PAACs in the α range 0.2–0.8 (*β*_1_) and 0.1−0.9 (*β*_2_).

Sample	*pKa*_1_^a,b^	*β*_1_^a^	*pKa*_2_^b,c^	*β*_2_^c^	IP ^d^
M-*l*-Ala	2.12 ± 0.04	0.76 ± 0.05	8.13 ± 0.17	1.32 ± 0.04	5.1
M-*l*-Val	2.08 ± 0.06	0.68 ± 0.09	6.78 ± 0.03	1.45 ± 0.08	4.4
M-*l*-Leu	2.11 ± 0.02	0.61 ± 0.07	7.37 ± 0.16	1.38 ± 0.07	4.7

^a^ Carboxyl group. ^b^ Average values obtained from four forward titration experiments. ^c^ Amine group. ^d^ Isoelectric point.

**Table 2:** Properties of the simulated PAACs at different pH values in water at the end of the MD runs and final geometry optimization.

Polymer	pH	Charge (*e*)	R_g_ *^a^* (nm)	*R ^b^* (nm)	*S ^c^* (nm^2^)	Volume *^d^* (nm^3^)	*μ ^e^* (D)
	1	+10	1.07	1.57	21.1	2.35	21.8
M-*l*-Ala	5	0	0.74	1.09	12.5	2.61	66.2
	11	−10	0.80	1.23	16.6	2.65	40.2
	1	+10	1.04	1.41	22.1	2.55	19.6
M-*l*-Val	5	0	0.73	1.57	13.2	2.80	20.6
	11	−10	0.85	1.46	19.9	2.66	38.1
	1	+10	1.11	1.73	24.1	2.70	24.4
M-*l*-Leu	5	0	0.85	0.84	19.9	2.95	43.0
	11	−10	0.80	1.14	16.9	3.11	28.0

*^a^* Gyration radius. *^b^* End to end Distance. *^c^* Surface area accessible to the solvent. *^d^* Volume of the optimized molecule in water. *^e^* Dipole moment.

**Table 3:** pH Dependence of PAAC hydrodynamic radius in water ***^a^***.

Polymer	*R_h_* (nm) pH 1.5	*R_h_* (nm) pH 5.0	*R_h_* (nm) pH 9.0
M-*l*-Ala	1.56 ± 0.52	1.54 ± 0.43	0.59 ± 0.17
M-*l*-Val	1.13 ± 0.33	0.90 ± 0.21	0.45 ± 0.17
M-*l*-Leu	1.40 ± 0.41	1.61 ± 0.49	0.67 ± 0.17

*^a^* Determined by DLS on polydisperse samples (see Experimental section) at 5 mg mL^−1^ concentration.

## Supplementary Materials

The following are available online at http://www.mdpi.com/2073-4360/10/11/1261/s1, Figure S1: ^1^H-NMR spectra recorded in D_2_O at pH = 4.5, Figure S2: FTIR-ATR spectra of the investigated PAACs, Figure S3: Forward and backward titration curves referred to the 1st experiment of Table S1, Figure S4: Determination of *β* parameters for side -COOH and chain *tert*-amine of M-*l*-Ala, M-*l*-Val and M-*l*-Leu referred to the 1st experiment of Table S1: calculation of *β* values from Equation (S1b) (a–c); trend of the *β*-corrected *pK_a_* values versus *α* according to Equation (S1a) (d–f), Figure S5: pH Dependence of circular dichroism spectra of (a) M-*l*-Ala, (b) M-*l*-Val and c) M-*l*-Leu at 25 °C, Figure S6: Comparison of the optimized geometry of M-*l*-Ala at pH 1 obtained in implicit water using the solvent dielectric constant (before simulations in explicit water) using the two simulation strategies described in the main text. In green the conformation obtained at 300 K, superimposed to the final geometry eventually obtained after the MD runs for 500 ps at 500 K, then for 500 ps at 400 K and finally 500 ps at 300 K (color codes: C atoms dark grey; H atoms light gray; N atoms blue; O atoms red) is shown. In the upper part of the figure, the superposition of the whole molecules is shown in CPK representation. The lower part reports the conformations of the main chain with no H atoms that were eventually achieved by the two strategies at 300 K viewed along three orthogonal directions; Table S1: PAAC *pK_a_* values from forward and backward titration data.
