# Supplementary material for: Self-Structuring in Water of Polyamidoamino Acids with Hydrophobic Side Chains Deriving from Natural α-Amino Acids
Source: Polymers (Basel). 2018 Nov 13;10(11):1261. doi: 10.3390/polym10111261 (PMC6401854; doi:10.3390/polym10111261)
Supplement: Supplementary file 1 [file polymers-10-01261-s001.pdf]

Supplementary Materials

Self-Structuring in Water of Polyamidoamino Acids with Hydrophobic Side Chains Deriving from Natural α-Amino Acids

Federica Lazzari [1], Amedea Manfredi [1], Jenny Alongi [1,*], Raniero Mendichi [2], Fabio Ganazzoli [3], Giuseppina Raffaini [3,*], Paolo Ferruti [1,*] and Elisabetta Ranucci [1,*]

[1] Dipartimento di Chimica, Università degli Studi di Milano, via C. Golgi 19, 20133 Milano, Italy; federica.lazzari@unimi.it (F.L.); amedea.manfred@unimi.it (A.M.)
[2] Istituto per lo Studio delle Macromolecole (CNR), Via E. Bassini 15, 20133 Milano, Italy; mendichi@ismac.cnr.it (R.M.)
[3] Dipartimento di Chimica, Materiali ed Ingegneria Chimica "G. Natta", Politecnico di Milano, via L. Mancinelli 7, 20131 Milano, Italy; fabio.ganazzoli@polimi.it (F.G.)

Correspondence: jenny.alongi@unimi.it; Tel.: +39-02-50314108 (J.A.); giuseppina.raffaini@polimi.it; Tel.: +39-02-23993058 (G.R.); paolo.ferruti@unimi.it; Tel.: +39-02-50314132 (P.F.); elisabetta.ranucci@unimi.it; Tel.: +39-02-50314132 (E.R.)

^{1}H-NMR and FTIR-ATR spectra

Determination of pK_a values and β parameters

Circular Dichroism (CD) spectra

Simulation method

Figures S1-S6

Tables S1

References

PAAC	^{1}H-NMR spectrum
**M-*L*-Ala**<br><br>δ (ppm) 1.40-1.43 (A), 2.64-2.79 (B), 3.24-3.42 (C), 3.90-3.96 (D), 4.54 (E).	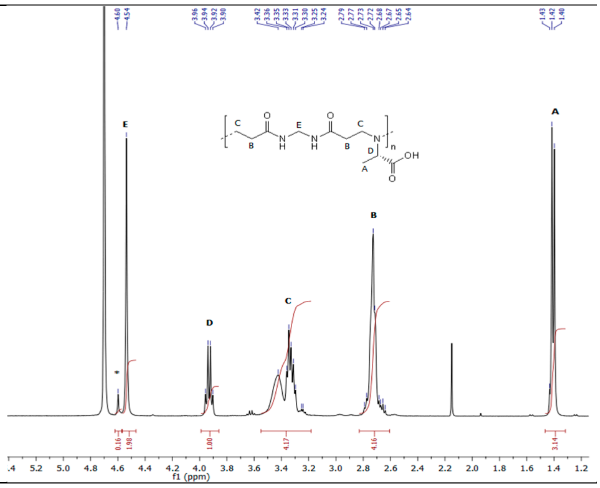
**M-*L*-Val**<br><br>δ (ppm) 0.92-0.93 (A), 1.04-1.05 (B), 2.23-2.25 (C), 2.67-2.75 (D), 3.44-3.50 (E, F), 4.53 (G).	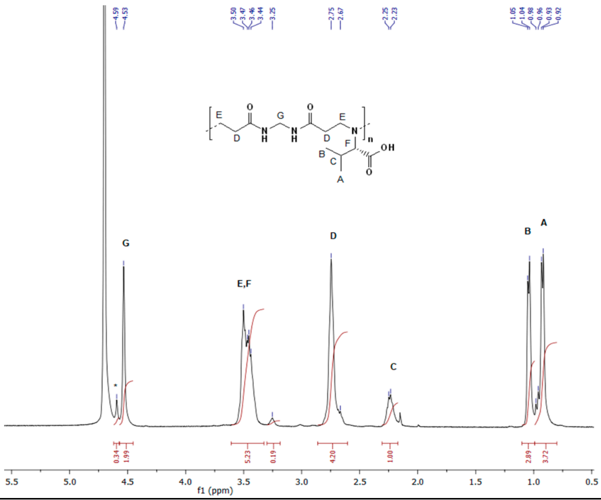
**M-*L*-Leu**<br><br>δ (ppm) 0.89 (A), 1.50-1.55 (B), 1.76-1.83 (C), 2.72-2.75 (D), 3.42-3.45 (E), 3.69-3.73 (F), 4.54 (G).	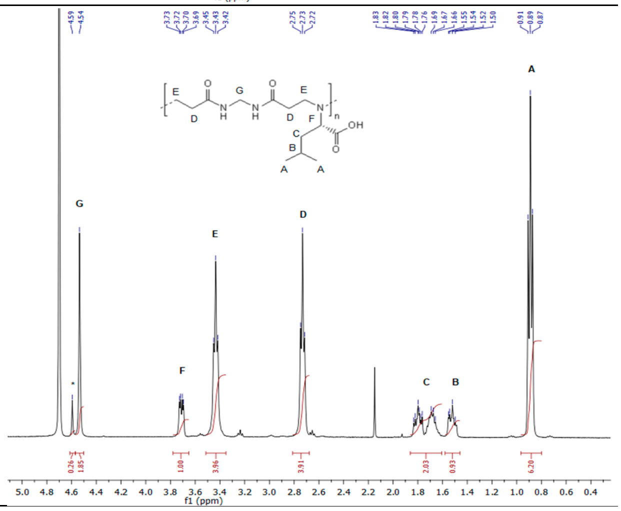

Figure S1. ^{1}H-NMR spectra recorded in D_2O at pH = 4.5.

Fourier-transform infrared spectroscopy (FTIR) analysis. FTIR in attenuated total reflectance configuration (ATR) spectra were recorded performing 16 scans at 4 cm^{-1} resolution in the 4000-500 cm^{-1} range, using a Perkin Elmer Spectrum 100 spectrometer equipped with a diamond crystal (penetration depth = 1.66 μm). Before each analysis, all samples were dried under vacuum to constant weight.

PAAC	FTIR-ATR spectrum

M-*L*-Ala

Signal	Wavenumber (cm^{-1})	Assignments
1	3259	N-H stretch
2	3045-2948	C-H stretch
3	1618	C=O stretch
4	1531	N-H bend
5	1357	O-H bend
6	1211	C-N stretch
7	1113	C-O stretch

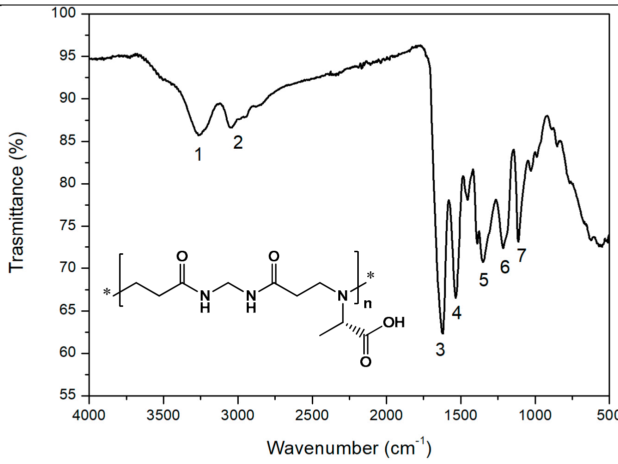

M-*L*-Val

Signal	Wavenumber (cm^{-1})	Assignments
1	3273	N-H stretch
2	3058-2873	C-H stretch
3	1631	C=O stretch
4	1537	N-H bend
5	1375	C-H bend
6	1326	O-H bend
6	1194	C-N stretch
7	1117	C-O stretch

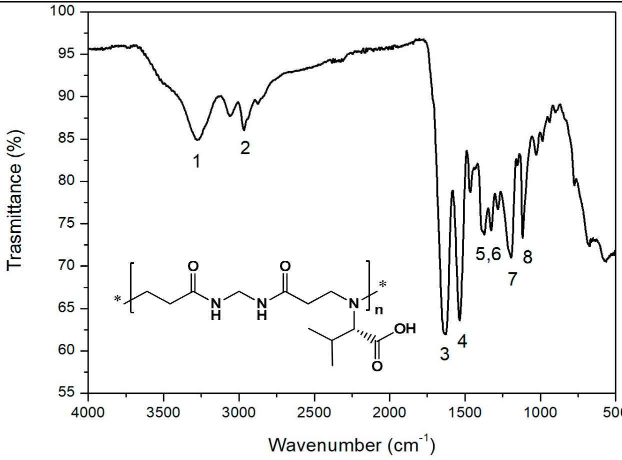

M-*L*-Leu

Signal	Wavenumber (cm⁻¹)	Assignments
1	3278	N-H stretch
2	3065-2871	C-H stretch
3	1639	C=O stretch
4	1532	N-H bend
5	1367	O-H bend
6	1192	C-N stretch
7	1115	C-O stretch

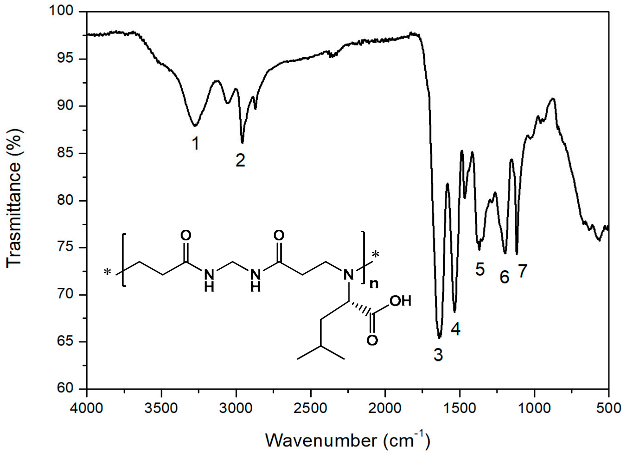

Figure S2. FTIR-ATR spectra of the investigated PAACs.

Determination of pKa values, *β* parameters and speciation curves

pKa determination. The pK_{a1} (side -COOH) and pK_{a2} (chain *tert*-amine) values of the ionizable functions present in the studied PAACs were determined as the pH values at the half-equivalent points, located in the buffer zone related to the specific function. The half-equivalent points were obtained as the half-titrant volume amounts added between consecutive inflections in the pH versus titrant volume curves. The inflection points were in turn determined by numerically calculating the second derivative of the pH versus volume curves (Fig. S3). The titration of the *tert*-amine group started around pH 5, that is, after complete deprotonation of the carboxyl group. Deprotonation of the ammonium ion took place between the first and second inflections. Therefore, the half-neutralization point was evaluated as the mid-point between them. In forward titrations, the starting pH was 1.8, that is, at incomplete COOH deprotonation. Therefore, in order to determine the midpoint of the COOH deprotonation tract, the starting of COOH deprotonation was extrapolated by considering that COOH and *tert*-amine groups were in equal numbers, hence COOH deprotonation needed the same titrant volume as that used for the ammonium ion deprotonation.

***β parameter determination*.** The *β* parameters of the generalized Henderson-Hasselbalch equation (Eq. S1a) were determined for both pK_{a1} (side -COOH) and pK_{a2} (chain *tert*-amine) to ascertain the presence of interactions between ionizable groups on adjacent monomeric units. The *β* parameters were determined by firstly selecting the specific buffer region intervals marked by each pK_a. The dissociation degree, *α*, was then calculated in each zone as the ratio between the reacted moles and the total amount of moles necessary to reach complete neutralization. *β* Values were finally obtained from Eq. S1b (corresponding to Eq. 2 in the main text) as the slope of the pH versus –log((1-

$\alpha)/\alpha)$ curve (Fig. S4). Points near inflections deviated from ideality and were not considered. Fig. S4 shows the β-corrected pK_a values in the chosen α intervals.

$$pK_a = pH + \beta\ log\frac{1-\alpha}{\alpha} \quad \text{(Eq. S1a)} \quad \leftrightarrow \quad pH = pK_a - \beta\ log\frac{1-\alpha}{\alpha} \quad \text{(Eq. S1b)}$$

Determination of simulated titration curves. Simulated titration curves were determined following the De Levie approach [1] in order to iteratively refine pKa and β values to achieve the best fitting to the experimental data.

- Initial conditions:

V_0 = initial solution volume

c_0 = initial PAACs concentration expressed as molarity of the repeat unit

c_S = initial concentration of ionic strength stabilizer

c_t = titrant concentration

V_t = volume of the titrant added

c_A = acid concentration used to correct pH

N = moles of strong acid possibly present as residual from the synthetic process or PAACs pretreatments

- *Mass balance:*

$$C_{PAACS} = C_{L^+} + C_{L^0} + C_{L^-} = \frac{C_0V_0}{V_0+V_t} \quad \text{(Eq. S2)}$$

- *Equilibrium constants* (Eq. S3a-c):

$$K_{a1} = \frac{C_{L^0}C_{H^+}}{C_{L^+}} \quad \text{(a);} \quad K_{a2} = \frac{C_{L^-}C_{H^+}y^2}{C_{L^0}} \quad \text{(b);} \quad K_w = C_{H^+}C_{OH^-}y^2 \quad \text{(c);}$$

- *Concentration fractions* (Eq. S4a-c):

$\alpha_2 = \frac{C_{L^+}}{C} = \frac{C_{H^+}^2}{D}$ (a); $\alpha_1 = \frac{C_{L^0}}{C} = \frac{C_{H^+} y^2 K_{a1}}{D}$ (b); $\alpha_0 = \frac{C_{L^-}}{C} = \frac{K_{a1} K_{a2}}{D}$ (c);

with:

$$D = C_{H^+}^2 + C_{H^+} K_{a1} + K_{a1} K_{a2} \qquad \text{(Eq. S5)}$$

The activity coefficients (Davies equation):

$$y = 10^{-0.5\left[\frac{\sqrt{I}}{1+\sqrt{I}} - 0.3I\right]} \qquad \text{(Eq. S6)}$$

Ionic strength:

$$I = \frac{1}{2}(C_{H^+} + C_{OH^-} + C_{Na^+} + C_{Cl^-} + C_{L^+} + C_{L^-}) \quad \text{(Eq. S7)}$$

- *Charge balance*:

$$H^+ + Na^+ + L^+ = L^- + OH^- + Cl^- \qquad \text{(Eq. S8)}$$

where (Eq. S9a-e):

$C_{Na^+} = \frac{C_T V_T + C_S V_0}{V_0 + V_T}$ (a); $C_{Cl^-} = \frac{C_S V_0 + C_A V_A + N}{V_0 + V_T}$ (b); $C_{L^+} = \frac{\alpha_2 C_0 V_0}{V_0 + V_T}$ (c);

$C_{L^-} = \frac{\alpha_0 C_0 V_0}{V_0 + V_T}$ (d); $C_{OH^-} = \frac{K_w}{C_{H^+} y^2}$ (e);

Combining all former conditions, the following solving equation, representing the whole forward titration curve, was obtained in terms of V_T as a function of pH:

$$V_T = \frac{V_0[C_0(\alpha_0 - \alpha_2) + C_A - \Delta] + N}{\Delta + C_T} \qquad \text{(Eq. S10)}$$

where:

$$\Delta = H^+ - OH^- = H^+ - {K_w}/{H^+y^2} \qquad \text{(Eq. S11)}$$

The best fitting simulated titration curves, obtained from Eq. S10 in the buffer regions relative to both side -COOH and *tert*-amine groups by using the iteratively refined *pKa* and β values reported in Table 1 of the main text, are shown in Fig. S3. Comparison between pK_a and β-corrected pK_a values is reported. Calculation were carried out considering C_{Na^+} and C_{Cl^-} constant throughout the whole titration experiment and equal to 0.1 M. Concentration fractions *a* and pK_a values were refined iteratively to achieve the best fitting to the experimental points.

Determination of speciation diagrams. Speciation diagrams were obtained by plotting the concentration fractions of the differently ionic species as a function of pH (Eq. S12a-c):

$$\alpha_2 = \frac{C_{L^-}}{C} = \frac{C_{H^+}^2}{D} \qquad \text{Eq. S12a}$$

$$\alpha_1 = \frac{C_{L^0}}{C} = \frac{C_{H^+}y^2K_{a1}}{D} \qquad \text{Eq. S12b}$$

$$\alpha_0 = \frac{C_{L^-}}{C} = \frac{K_{a1}K_{a2}}{D} \qquad \text{Eq. S12c}$$

With D and y as previously described, and where the K_{a1} and K_{a2} values were corrected for β_1 and β_2.

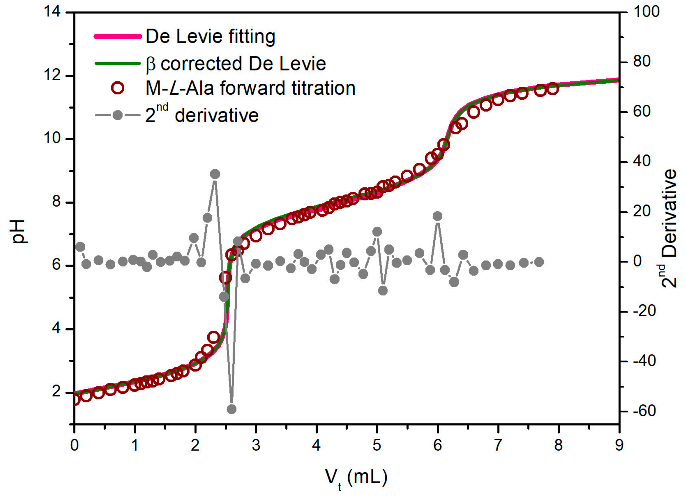
De Levie fitting
β corrected De Levie
M-L-Ala forward titration
2nd derivative
pH
2nd Derivative
Vt (mL)

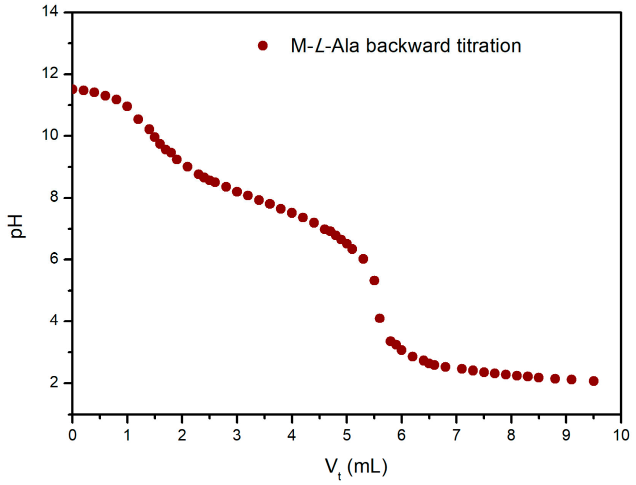
M-L-Ala backward titration
pH
Vt (mL)

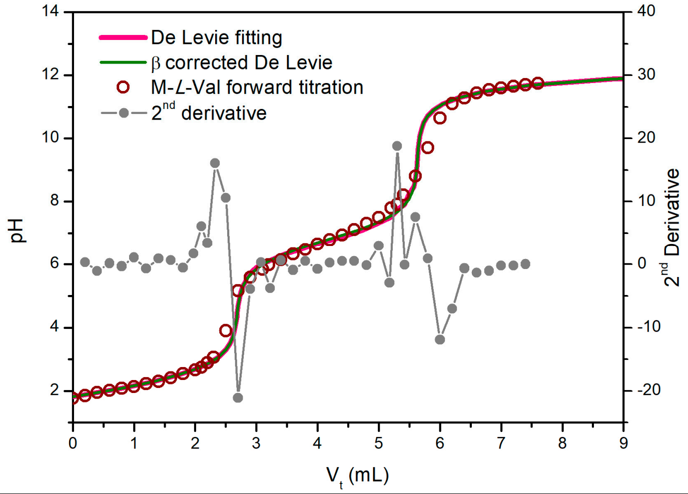
De Levie fitting
β corrected De Levie
M-L-Val forward titration
2nd derivative
pH
2nd Derivative
Vt (mL)

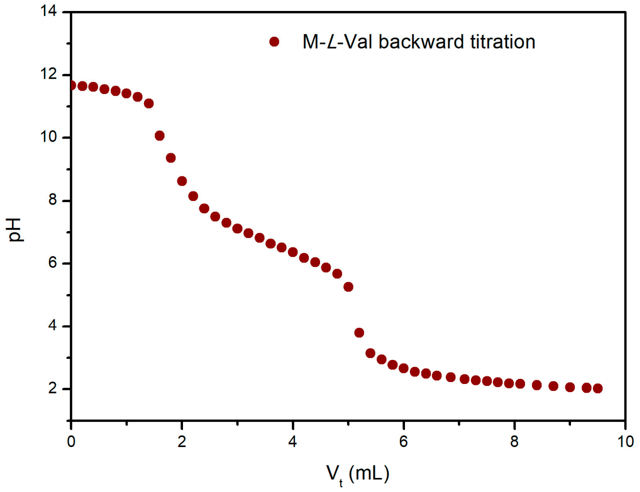
M-L-Val backward titration
pH
Vt (mL)

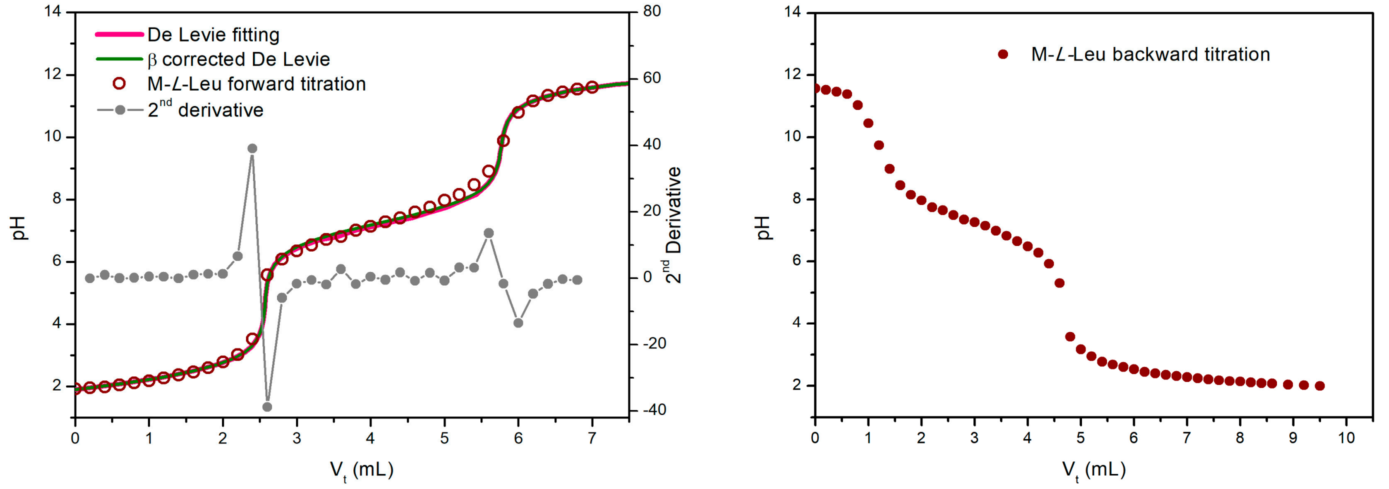

Figure S3. Forward and backward titration curves referred to the 1st experiment of Table S1.

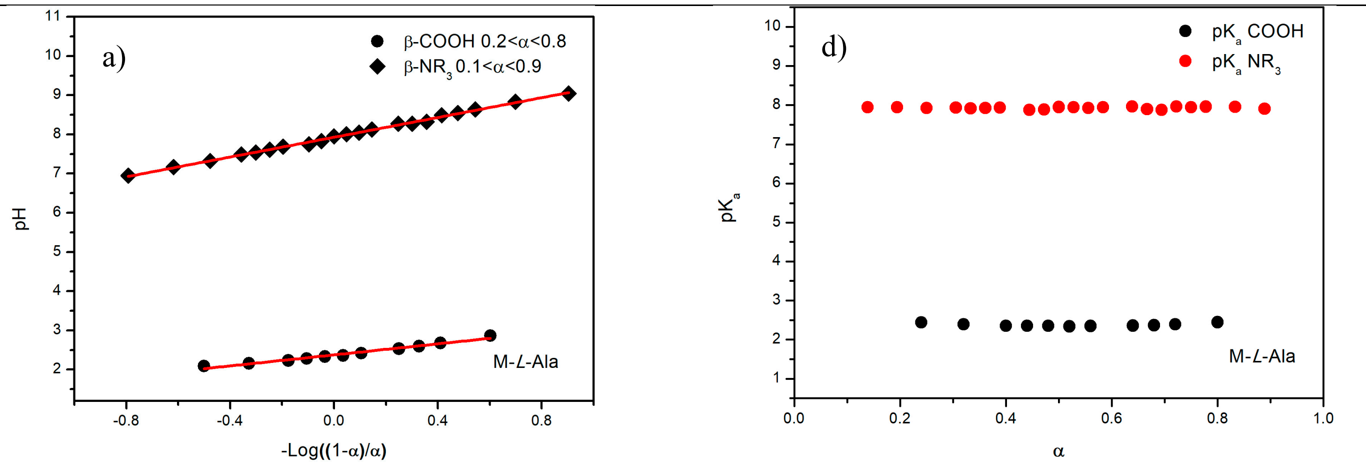

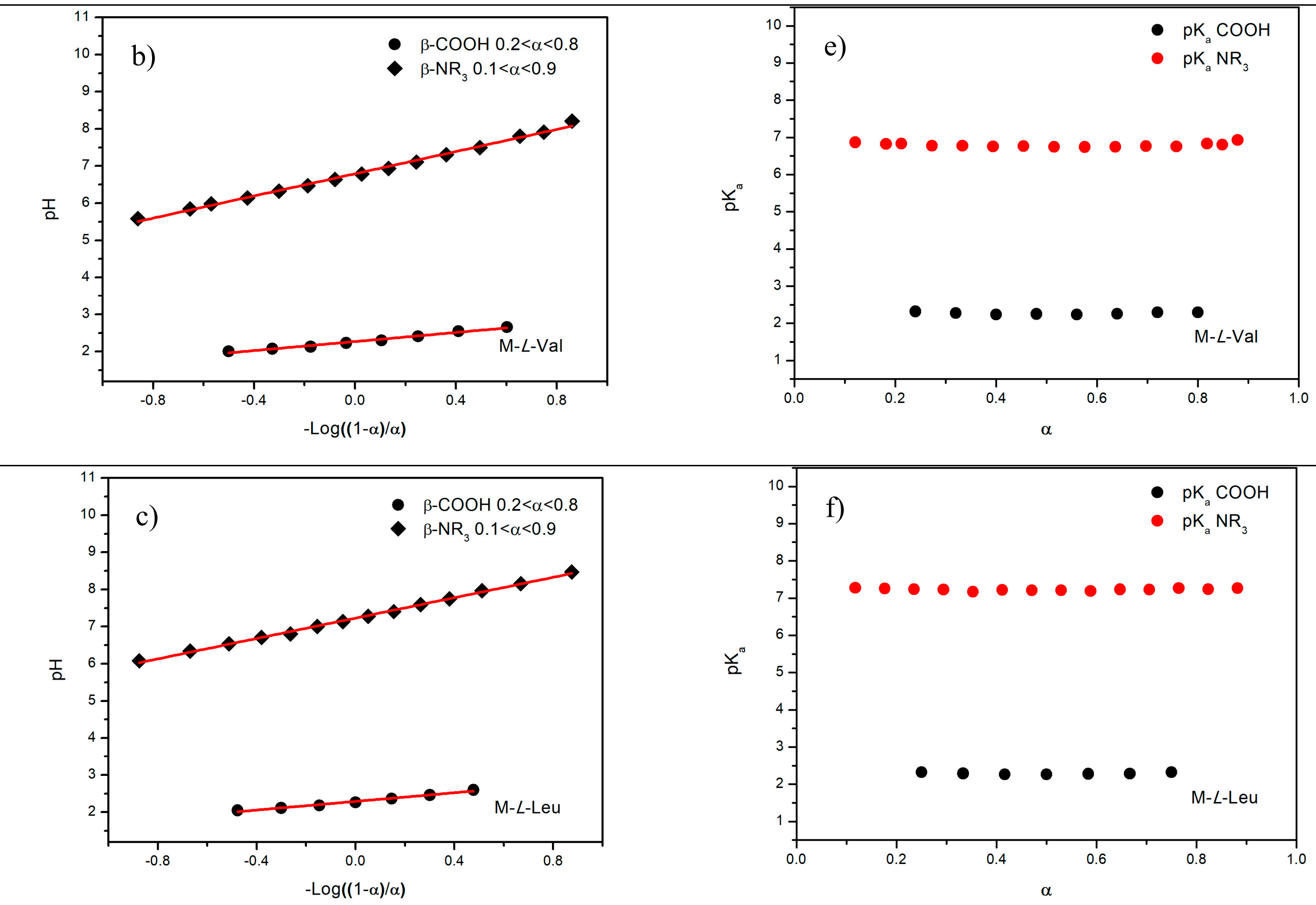

Figure S4. Determination of β parameters for side -COOH and chain *tert*-amine of M-*L*-Ala, M-*L*-Val and M-*L*-Leu referred to the 1st experiment of Table S1: calculation of β values from Eq. 1b (a, b, c); trend of the β-corrected pK_a values versus a according to Eq. S1a (d, e, f).

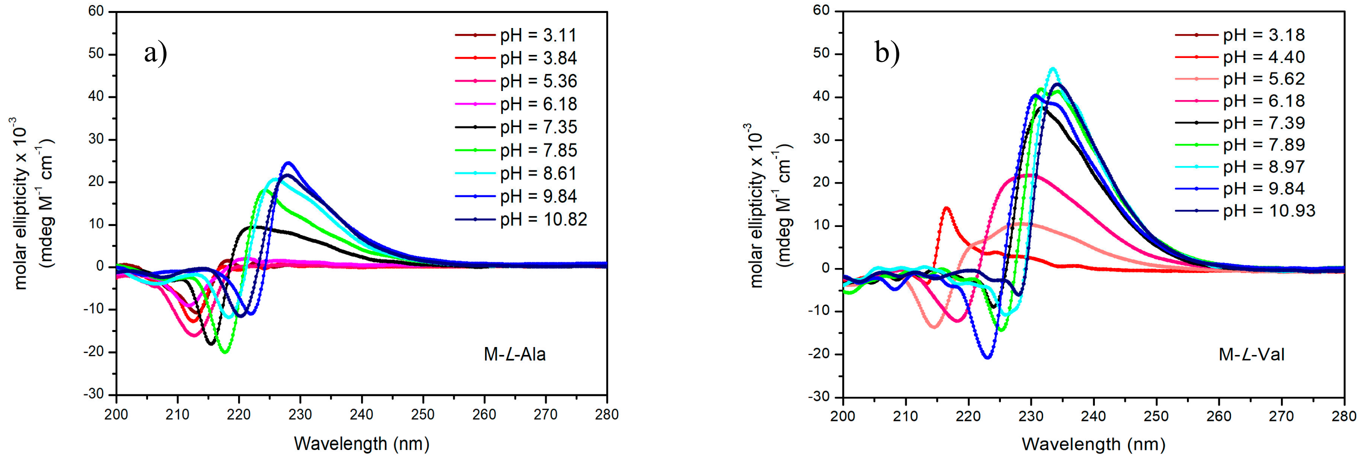

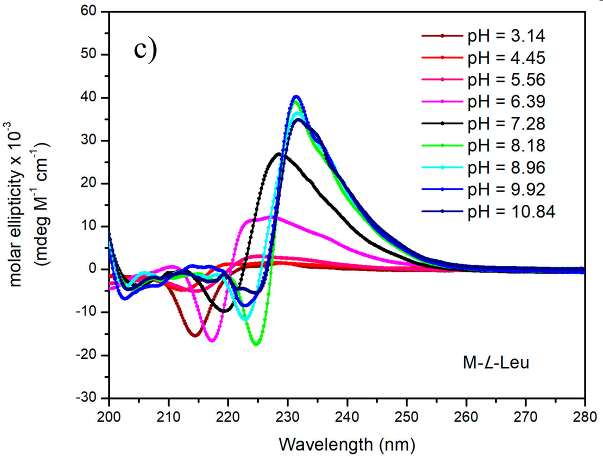

Figure S5. pH Dependence of circular dichroism spectra of a) M-*L*-Ala, b) M-*L*-Val and c) M-*L*-Leu at 25 °C.

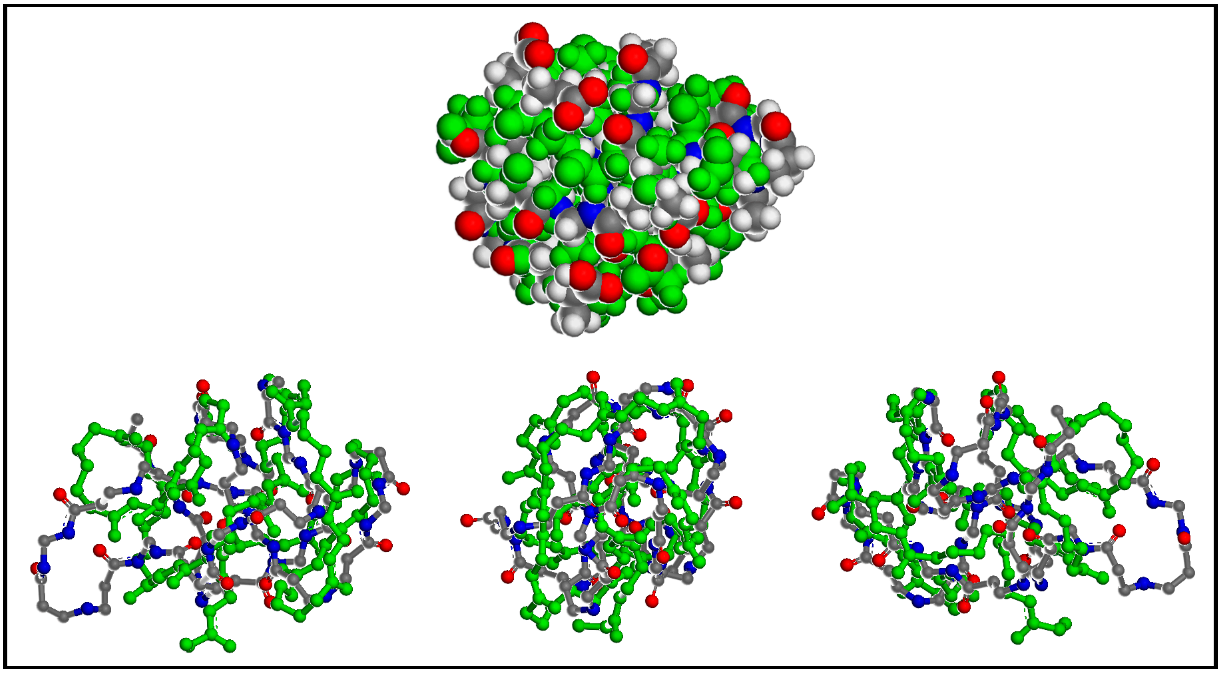

Figure S6. Comparison of the optimized geometry of M-*L*-Ala at pH 1 obtained in implicit water using the solvent dielectric constant (before simulations in explicit water) using the two simulation strategies described in the main text. In green the conformation obtained at 300 K, superimposed to the final geometry eventually obtained after the MD runs for 500 ps at 500 K, then for 500 ps at 400 K and finally 500 ps at 300 K (color codes: C atoms dark grey; H atoms light gray; N atoms blue; O atoms red) is shown. In the upper part of the figure, the superposition of the whole molecules is shown in CPK representation. The lower part reports the conformations of the main chain with no H atoms that were eventually achieved by the two strategies at 300 K viewed along three orthogonal directions.

Table S1. PAAC pK_a values from forward and backward titration data.

				**M-*L*-Ala**				
**Forward**	1st		2nd		3rd		4th	
	-COOH	$-NR_3$	-COOH	$-NR_3$	-COOH	$-NR_3$	-COOH	$-NR_3$
pK_a	2.07	7.90	2.15	8.27	2.13	8.15	2.13	8.24
**Backward**	1st		2nd		3rd		4th	
	-COOH	$-NR_3$	-COOH	$-NR_3$	-COOH	$-NR_3$	-COOH	$-NR_3$
pK_a	2.37	7.77	2.36	8.22	2.35	8.18	2.37	8.22
				**M-*L*-Val**				
**Forward**	1st		2nd		3rd		4th	
	-COOH	$-NR_3$	-COOH	$-NR_3$	-COOH	$-NR_3$	-COOH	$-NR_3$
pK_a	2.12	6.78	1.98	6.74	2.11	6.77	2.10	6.82
**Backward**	1st		2nd		3rd		4th	
	-COOH	$-NR_3$	-COOH	$-NR_3$	-COOH	$-NR_3$	-COOH	$-NR_3$
pK_a	2.37	6.86	2.22	6.78	2.35	6.79	2.41	6.92
				**M-*L*-Leu**				
**Forward**	1st		2nd		3rd		4th	
	-COOH	$-NR_3$	-COOH	$-NR_3$	-COOH	$-NR_3$	-COOH	$-NR_3$
pK_a	2.14	7.23	2.13	7.45	2.09	7.24	2.10	7.55
**Backward**	1st		2nd		3rd		4th	
	-COOH	$-NR_3$	-COOH	$-NR_3$	-COOH	$-NR_3$	-COOH	$-NR_3$
pK_a	2.37	7.16	2.42	7.28	7.28	2.40	7.23	2.68

Simulation method

The simulation method and the adopted strategy exactly matched those described in ref. [20] of the main text and are briefly summarized here. The InsightII/Discover 2000 [2] package of programs was used adopting the consistent valence force field CVFF [3]. The molecules, comprising 10 repeat units, were prepared using the available templates assuming the appropriate charges at different pH values in order to have a positive (L^+), a null (L^0) and a negative (L^-) charge per repeat unit.

Starting from a fully extended chain, after an initial geometry optimization, the molecules were subjected to MD runs of 5 ns in an effective medium with a distance-dependent dielectric constant, and then of 0.5 ns in explicit water, adopting a cubic cell with a local density of 1 g cm^{-3}, with final geometry optimizations. Equilibration of the system was monitored through the time change of the total and potential energy together with its components (such as the Coulomb and the van der Waals and the torsional components), and of the end-to-end distance. The dynamic equations were integrated using the Verlet algorithm with a time step of 1 fs at a temperature of 300 K, controlled through the Berendsen thermostat, and the instantaneous coordinates were periodically saved for further analysis. All the energy minimizations where carried out with the conjugate gradient method up to an energy gradient lower than 4×10^{-3} kJ mol^{-1} $Å^{-1}$.

References

[1] De Levie, R. *How to Use ExcelW in Analytical Chemistry and in General Scientific Data Analysis*; Cambridge University Press: Cambridge, 2001.

[2] Accelrys Inc. InsightII 2000 and Materials Studio: San Diego, CA. See also the URL http://www.accelrys.com.

[3] Dauber-Osguthorpe, P.; Roberts, V.A.; Osguthorpe, D.J.; Wolff, J.; Genest, M.; Hagler, A.T. Structure and energetics of ligand binding to proteins: Escherichia coli dihydrofolate reductase-trimethoprim, a drug-receptor system. *Proteins* **1988**, *4*, 31–47.
